# T-cell infiltration in autosomal dominant neovascular inflammatory vitreoretinopathy

**Published:** 2010-06-08

**Authors:** Vinit B. Mahajan, John G. Vallone, Jonathan H. Lin, Robert F. Mullins, Audrey C. Ko, James C. Folk, Edwin M. Stone

**Affiliations:** 1 Vitreoretinal Service, Department of Ophthalmology and Visual Sciences, The University of Iowa Hospitals & Clinics; Iowa City, IA; 2 Omics Laboratory, Iowa City, IA; 3 Department of Pathology University of California San Diego; San Diego, CA; 4 Department of Pathology, University of California Irvine; Irvine, CA; 5 Howard Hughes Medical Institute, Iowa City, IA

## Abstract

**Purpose:**

Autosomal dominant neovascular inflammatory vitreoretinopathy (ADNIV) is a familial blinding disease of unknown pathophysiology. The eyes and sera from patients with ADNIV were studied to understand the immune response in this condition.

**Methods:**

The clinical case of an ADNIV patient was reviewed. Eye specimens from two donors with ADNIV were examined with a panel of standard histopathological stains and immunohistochemical markers. These findings were compared to specimens of noninflammatory eye disease. Sera from twelve patients were also tested against retinal protein western blots for the presence of autoretinal antibodies.

**Results:**

Each of the ADNIV and control eyes showed degenerative features of phthisis bulbi. Immunohistological stains revealed a supraciliary T-cell infiltrate in ADNIV eyes composed of cluster of differentiation-4 (CD4) positive and cluster of differentiation-8 (CD8)-positive cells. No immunoglobulin or B cells were detected in these eyes. Inflammatory cells were absent from the control eyes. No specific autoretinal antibodies were detected in ADNIV sera.

**Conclusions:**

Aberrant T-cell-mediated processes may underlie ADNIV, and therapeutics directed at T cells may better manage inflammation in these patients. Genes related to T-cell function are high priority screening candidates.

## Introduction

The eye is an immune-privileged site where the local immunological mechanisms are poorly understood. Autosomal dominant neovascular inﬂammatory vitreoretinopathy (ADNIV) is an autoimmune disease of the eye without systemic features [1,2]. ADNIV shares several features with more common vitreoretinal diseases, including diabetic retinopathy, idiopathic uveitis, proliferative vitreoretinopathy, and retinitis pigmentosa.

ADNIV is an eye-speciﬁc inﬂammatory condition characterized by pigmentary retinal degeneration, loss of the electroretinogram b-wave, and peripheral field loss [1]. This progressive degeneration is complicated by anterior segment and vitreous inflammation, retinal neovascularization, retinal detachment, and eventual phthisis. Cellular infiltrates in the vitreous are among the earliest detectable signs of ADNIV and continue throughout the course of the disease. The nature of the cells is not known.

Despite photoreceptor degenerative changes, one hypothesis suggests that ocular autoimmunity is the primary pathogenic cause of ADNIV and that the cells are either of B-cell or T-cell origin. Despite organ atrophy in the late stages of disease, antigens that instigate autoimmune reactions may still be active. The ADNIV autoimmune reaction continues through end-stage disease when the eye becomes shrunken and blind, and studies in these eyes may still be relevant to earlier stages of ADNIV. To better understand ADNIV pathogenesis, we performed studies to detect autoretinal antibodies and in the case of ADNIV autopsy eyes detect the presence of B-cell and T-cell infiltration.

## Methods

Informed consent was obtained to review the case history of a 80-year-old ADNIV patient examined in the University of Iowa Department of Ophthalmology clinic. The case history was reviewed for a first-generation ADNIV patient. We used six postmortem eyes (University of Iowa, Department of Pathology archived tissue collection), that had been received in formalin and post fixed in Pen-fix (Thermoscientific, Waltham, MA). After the eye was opened by pupil–optic nerve section, it was decalcified. Histological staining with Masson's Trichrome stain was performed according to the manufacturer’s protocol (Sigma-Aldrich, St. Louis, MO). Immunohistochemical staining was performed as follows. All slides were stained on the DAKO Autostainer+ (Carpinteria, CA), using heat pretreatment with a pressure cooker. All antibodies used Targert Retrieval pH 6.0 (#S1699; DAKO), except cluster of differentiation-4 (CD4), which used high-pH retrieval (#S3308; DAKO). The following antibodies were used: anti-CD3 (#A0452; DAKO) diluted to 1:200; anti-CD4 (#NCL-L-CD4–1F6; LeicaSystems, Bannockburn, IL) diluted to 1:40; anti-CD8 (#M7103; DAKO) diluted to 1:1,000; anti-CD20 (#M0755; DAKO) diluted to 1:400; anti-CD68 (#M0814; DAKO) diluted to 1:400; and anti-immunoglobulin G (IgG; #A0424; DAKO) diluted to 1:40,000. All antibodies were incubated for 30 min. A dual endogenous enzyme block (DAKO #S2003, Carpinteria, CA) was used for 5 min. Detection was for 30 min and DAB+ (DAKO #K3467, Carpinteria, CA) was used for 5 min. DAKO Envision+ Dual-Link labeled polymer (#K4061) was used for detection.

### Autoretinal antibody assay

Following informed consent, serum was collected from 12 patients with ADNIV (2 males, and 9 females; age range 7–68) and 12 unaffected, healthy controls (3 males, and 8 females; age range 18–74). The samples were screened on human retinal lysate to determine whether these sera contained autoantibodies against retinal antigens. Methods for western blot were performed, essentially as described previously [3]. Briefly, human retinal lysate was pooled from three donor eyes, separated by sodium dodecyl sulfate PAGE, and transferred to polyvinyldifluoride membrane. Serum from ADNIV patients or unaffected control patients without retinal disease was incubated with membrane strips to detect retinal antigens and visualized using horseradish peroxidase-conjugated antihuman secondary antibody. Results were compared between ADNIV patients and controls.

## Results

### Case report

An 80-year-old female originally presented with idiopathic posterior uveitis and retinitis pigmentosa. She underwent intracapsular cataract extractions 11 years prior and had postoperative visual acuity in the 20/200 range. Her right eye became phthisical 2 years before presentation, at which time she had a severe uveitis and vitreous hemorrhage in her left eye. She had no systemic inflammatory diseases, and a posterior uveitis workup was negative. Her family history suggested a genetic etiology, and she was found to be related to the original ADNIV pedigree that we first described in 1990 with similar clinical findings and genetic linkage to chromosome 11q13 [1,2].

**Figure 1:**
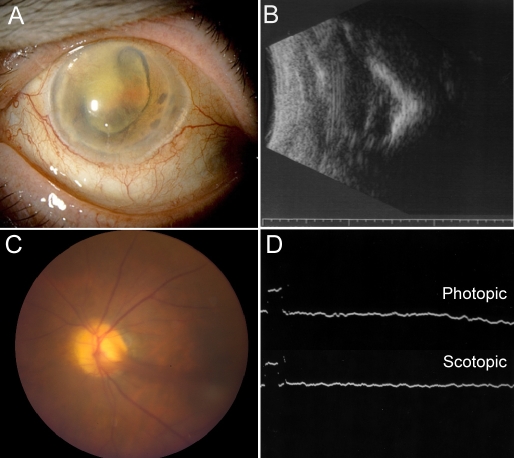
End-stage autosomal dominant neovascular inflammatory vitreoretinopathy. **A**: This phthisical right eye shows cloudy corneas, iris neovascularization, and pupillary fibrous membrane with aphakia. **B**: The B-scan demonstrates a foreshortened disorganized eye with total retinal detachment and calcification. **C**: The fellow prephthisical eye showed a neovascular stalk on the optic disc. **D**: Electroretinogram reveals extinguished photopic and scotopic responses.

On examination her visual acuity was no light perception right eye and light perception left eye. The intraocular pressures were 5 and 18 mm Hg, respectively. The anterior segment examination showed fulminant iris neovascularization. The right eye was phthisical with no view to the posterior pole (Figure 1A). Echography showed reduced axial length and small size along with a shell-like calcification of the choroid and dense vitreous opacities with extensive retinal detachment (Figure 1B). The left fundus revealed vitreous hemorrhage, pigmentary retinopathy, and peripheral neovascularization and fibrotic changes (Figure 1C). An electroretinogram of the left eye showed completely extinguished photopic and scotopic response (Figure 1D). Two years later, the left eye went into phthisis, and echography revealed tractional retinal detachment and posterior calcification. These clinical findings were characteristic of end-stage disease in several members of the ADNIV pedigree. When the patient expired, a pathological examination of the eyes was performed.

### Pathology findings

Gross examination of the eyes revealed shrunken, firm, grossly distorted globes. There were significant corneal opacities, collapsed anterior chambers, and a gray-brown, irregular, firm material within the vitreous cavities. The retinas and the choroids were detached, with accumulation of suprachoroidal fluid.

**Figure 2:**
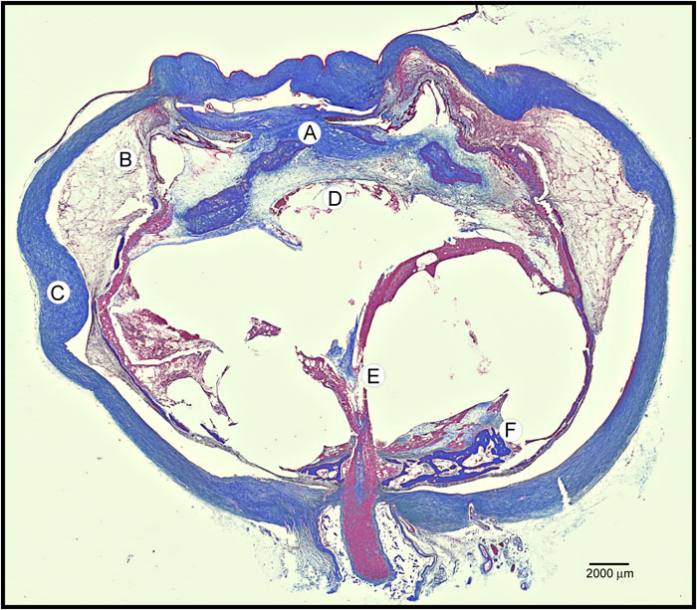
Masson’s trichrome stain of autosomal dominant neovascular inflammatory vitreoretinopathy (ADNIV) eye demonstrates fibrovascular proliferation and features of phthisis. **A**: There was massive fibrovascular connective tissue within the pupil, overlying the iris, and causing detachment of the ciliary body. **B**: A supraciliary effusion was present outside the detached ciliary body. **C**: The sclera was thickened. **D**: There were a few lens remnants present. **E**: A fibrovascular stalk extended from the optic nerve to the peripheral retina. **F**: The retina was detached, atrophic, and osseous metaplasia was present.

Typical features of phthisis bulbi were apparent on pupil–optic nerve sections (Figure 2). The cornea and sclera were thick, forming a shrunken square outline. The intraocular contents were grossly disorganized. An anterior hyaloid fibrovascular connective tissue detached the ciliary body, with underlying suprachoroidal effusions. The membrane extended over the iris and into a collapsed anterior chamber. The retinas were atrophic, cystic, and completely detached. A fibrovascular stalk extended from the disc anteriorly through a vitreous filled with proteinacious exudates (congo red negative, not shown). Osseous metaplasia was present along the retinal pigment epithelium and around atrophic optic nerves. These features were also present in control eyes that were phthisical from glaucoma, a noninflammatory condition without continuous autoimmunity found in ADNIV.

### Autoretinal antibodies

**Figure 3:**
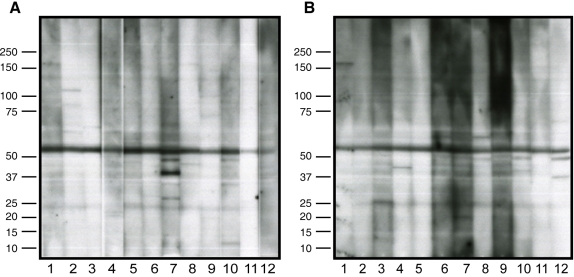
Antiretinal autoantibodies. **A**: Control serum from 12 subjects was applied to human retinal proteins separated by sodium dodecyl sulfate PAGE. Nonspecific banding patterns were detected. **B**: Serum from 12 autosomal dominant neovascular inflammatory vitreoretinopathy patients was applied to similar retinal blots, and no specific bands or unique patterns were detected. The major band in all samples indicates cross-reaction of secondary antibody with endogenous human immunoglobulin G (IgG) chains.

To determine whether systemic autoretinal antibodies were present in ADNIV patients, serum antibodies were applied to retinal protein blots. Sera from 12 control patients without ADNIV and 12 patients with ADNIV were compared (Figure 3). Although some immunoreactive bands were present in both the ADNIV and unaffected sera, no consistent distinct protein band or pattern of bands was present in the ADNIV subjects when compared to control samples.

### B-cell and immunoglobulin G immunohistopathology

**Figure 4:**
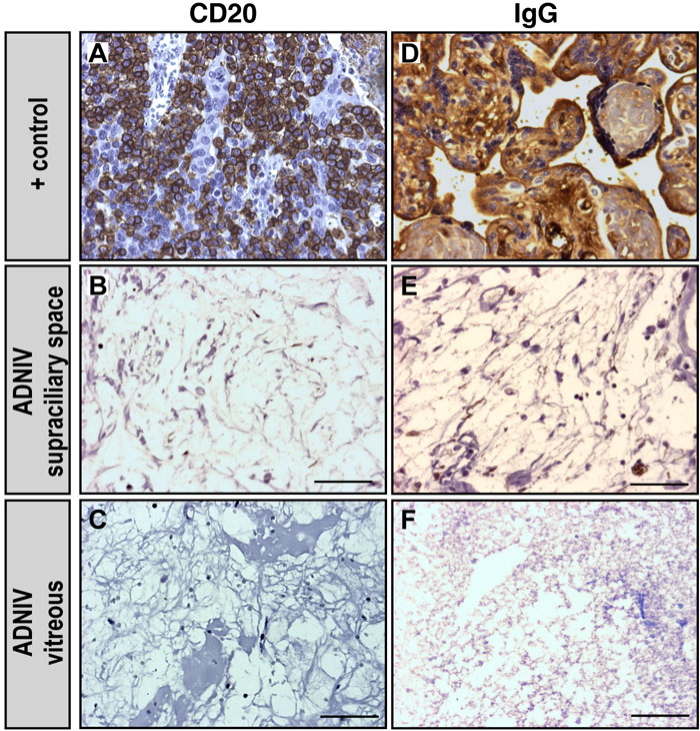
B-cell markers were absent in autosomal dominant neovascular inflammatory vitreoretinopathy (ADNIV) eyes. **A**: Lymph node tissue is a positive control for the B-cell marker, cluster of differentiation-20 (CD20), which was shown by the brown color reaction. **B**-**C**: ADNIV supraciliary space and vitreous showed only rare CD20 positive cells. **D**: Placental tissue is a positive control for immunoglobulin-G (IgG), which was shown by the brown color reaction. **E**-**F**: IgG was rarely detected in ADNIV eyes. The scale bar represents 50 µm.

A series of immunohistochemical markers for immune cells was applied to the ADNIV eyes. Antibody markers for both CD20 (a marker for B lymphocytes) and IgG were applied to the ADNIV eyes, but no CD20 cells or clusters of IgG were detected (Figure 4). These markers were also not seen in the control phthisis eyes.

### T-cell immunohistopathology

**Figure 5:**
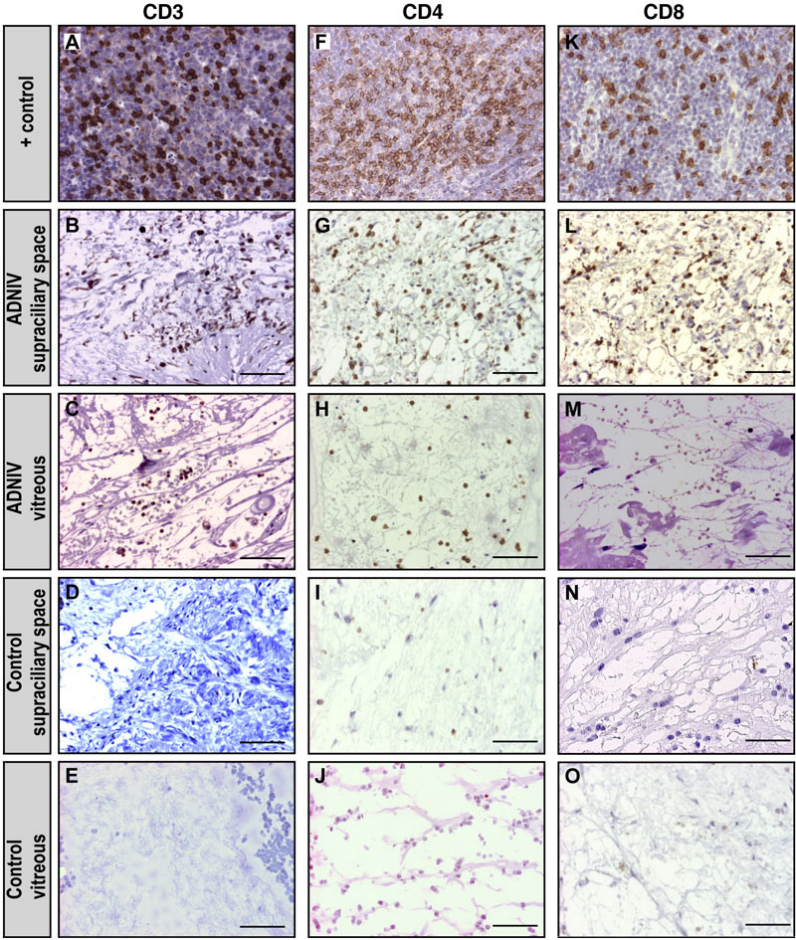
T-cell markers were detected in autosomal dominant neovascular inflammatory vitreoretinopathy (ADNIV) eyes. **A**: Lymph node tissue is a positive control for the T-cell marker, cluster of differentiation-3 (CD3), which was shown by the brown color reaction. **B**-**C**: In ADNIV eyes, the supraciliary space and vitreous showed CD3 positive cells. **D**-**E**: In control (non-ADNIV) phthisical eyes, CD3 positive cells were absent. **F**: Lymph node tissue is a positive control for the T-cell marker, cluster of differentiation-4 (CD4), which was shown by the brown color reaction. **G**-**H**: In ADNIV eyes, the supraciliary space and vitreous both showed CD4 positive cells. **I**-**J**: In control (non-ADNIV) phthisical eyes, CD4 positive cells were absent. **K**: Lymph node tissue is a positive control for the T-cell marker, cluster of differentiation-8 (CD8), which was shown by the brown color reaction. **L**: In ADNIV eyes, the supraciliary space showed CD8 positive cells. **M**: In the ADNIV vitreous, CD8 positive cells were absent. **N**-**O**: In control (non-ADNIV) phthisical eyes, CD8 positive cells were absent. The scale bar represents 50 μm.

In the ADNIV eyes, CD3-positive (CD3^+^) cells were detected within a supraciliary effusion (Figure 5). Such an effusion was not present in the control eyes. CD3^+^ cells were also present in the vitreous. To distinguish these T cells, antibody markers for CD8 and CD4 were applied. Both CD8^+^ and CD4^+^ cells were present in the supraciliary effusion, but only CD4^+^ cells were found in the vitreous.

The macrophage marker CD68 was detected infrequently and did not show expression differences between ADNIV and control eyes (data not shown).

## Discussion

These findings support the hypothesis that an underlying ocular immune dysfunction is present in ADNIV. CD4 expression, which is found predominantly on the surface of helper T cells, was found in both the vitreous and supraciliary space. CD8 expression, which is found predominantly on the surface of cytotoxic T cells, was mostly restricted to the supraciliary space. By comparison, there were few B cells, IgG, and no specific antiretinal autoantibodies. This suggests that cell-mediated rather than antibody-mediated autoimmunity may be the primary inflammatory mechanism.

The genetic locus for ADNIV was mapped to chromosome 11q13 in 1992 [2], but the causative gene remains unknown. These findings suggest genes that function in T-cell activation could be prioritized in candidate gene sequencing. For example, one gene in the interval is tumor necrosis factor (TNF) receptor superfamily member 19 (TNFRSF19L), a member of the TNF receptor superfamily, which can stimulate T-cell proliferation in the presence of CD3 signaling [4]. Until the causative gene is found and highly specific therapy can be applied, current management of ADNIV involves local immunosuppression with periocular or intraocular Kenalog. This nonspecific therapy might be optimized by medications directed at the specific mediators of ADNIV. Our findings suggest that therapeutic strategies targeting T cells may be more effective than nonspecific therapies or those that target B cells. For example, drugs, such as cyclosporin A, FK06, anti-CD3, and rapamycin, that target T-cell activation and downstream T-cell pathways might have greater effect than B-cell drugs, such as cyclophosphamide, mycophenylate mofetil, anti-inducible costimulatory molecule (ICOS), anti-CD20, or anti-CD52 [5]. Like some other uveitic conditions, ADNIV eventually becomes resistant to conventional steroid immunosuppression. It is possible that steroid refractory T cells are present, and therapies directed at steroid-independent mechanisms may overcome this limited response [6].

The main limitation of this study is the few number of eyes, but ADNIV is rare and there are no previous histopathological reports. The eyes in this study were phthisical and represent late stage ADNIV. Nevertheless, there is an ongoing autoimmune reaction in this disease, and phthisis in the ADNIV eye seems to be immunologically different from that observed in control eyes with phthisis from other conditions [7,8]. This study provides a basis for future studies that will test vitreous samples from early stage ADNIV to confirm the presence of T cells [9].
